# Risk of Covid-19 Severe Outcomes and Mortality in Migrants and Ethnic Minorities Compared to the General Population in the European WHO Region: a Systematic Review

**DOI:** 10.1007/s12134-023-01007-x

**Published:** 2023-01-11

**Authors:** Elena Mazzalai, Dara Giannini, Maria Elena Tosti, Franca D’Angelo, Silvia Declich, Anissa Jaljaa, Susanna Caminada, Federica Turatto, Chiara De Marchi, Angela Gatta, Aurora Angelozzi, Giulia Marchetti, Scilla Pizzarelli, Maurizio Marceca

**Affiliations:** 1 Department of Public Health and Infectious Diseases, Sapienza University of Rome, Rome, Italy; 2 Italian Society of Migration Medicine (SIMM), Rome, Italy; 3 National Centre for Global Health, Istituto Superiore di Sanità, Rome, Italy; 4 Department for Organisational Development, Local Health Unit Roma 1, Rome, Italy; 5 Knowledge Unit, Documentation and Library, Istituto Superiore di Sanità, Rome, Italy

**Keywords:** Covid-19, Ethnic minorities, Migrants, Severe outcomes, Europe, Health inequalities

## Abstract

The Covid-19 pandemic has had a major impact on migrants and ethnic minorities (MEMs). Socio-economic factors and legal, administrative and language barriers are among the reasons for this increased susceptibility. The aim of the study is to investigate the impact of Covid-19 on MEMs compared to the general population in terms of serious outcomes. We conducted a systematic review collecting studies on the impact of Covid-19 on MEMs compared to the general population in the WHO European Region regarding hospitalisation, intensive care unit (ICU) admission and mortality, published between 01/01/2020 and 19/03/2021. Nine researchers were involved in selection, study quality assessment and data extraction. Of the 82 studies included, 15 of the 16 regarding hospitalisation for Covid-19 reported an increased risk for MEMs compared to the white and/or native population and 22 out of the 28 studies focusing on the ICU admission rates found an increased risk for MEMs. Among the 65 studies on mortality, 43 report a higher risk for MEMs. An increased risk of adverse outcomes was reported for MEMs. Social determinants of health are among the main factors involved in the genesis of health inequalities: a disadvantaged socio-economic status, a framework of structural racism and asymmetric access to healthcare are linked to increased susceptibility to the consequences of Covid-19. These findings underline the need for policymakers to consider the socio-economic barriers when designing prevention plans.

**Supplementary Information:**

The online version contains supplementary material available at 10.1007/s12134-023-01007-x.

## Background

With more than 600 million cases and over 6 million deaths, as of 7th October 2022, the Covid-19 pandemic represents an unprecedented challenge for countries and health systems worldwide (WHO, 2022a, 2022b). Along with a high toll in terms of death and health outcomes in the population as a whole, the Covid-19 pandemic has also had a recognised impact in widening health inequalities, both between and within countries (Bambra, 2020; Lau, 2020; Marmot & Allen, 2020). Socially disadvantaged population groups have experienced particular vulnerability to the pandemic (Patel JA, 2020).

Migrants and ethnic minorities (MEMs) in particular have been shown to suffer a higher burden both in terms of risk of infection and in terms of negative health outcomes (Greenway, 2020; Hayward, 2021; Phiri, 2021; Sze, 2020). The mechanisms underlying the higher susceptibility of MEMs to worse health outcomes of Covid-19 include factors already known to create health inequity, such as socio-economic difficulty barriers to accessing healthcare such as legal, administrative, linguistic and cultural barriers, in addition to a higher prevalence of pre-existing conditions, often neglected (Greenway, Marceca, 2017; Shaaban, 2020; Stevens, 2021; Woodward, 2014).

While there is growing evidence on the differential impact of diseases on MEMs worldwide, data specific to the WHO European Region is scarce, partly due to the difficulty of retrieving data on ethnicity and migration background for public health purposes (Bozorgmehr, 2019; Corvacho, 2021; Melchior, 2021). The issue of the health of MEMs in the region, however, is pressing, since the WHO European Region is a very ethnically diverse territory and it is estimated that almost 10% of the population living in the WHO European Region is made up of migrants, therefore making clearer that there is no public health without refugee and migrant health (WHO, 2010; WHO, 2018; Abubakar et al., 2018).

In a previously published systematic review (Jaljaa, 2022), we have analysed the higher risk of infection for MEMs in the WHO European Region. The aim of this paper is to collect and synthesise evidence on the differential impact of Covid-19 on MEMs compared to the general population in the WHO European Region, in terms of negative health outcomes, considering risk of hospitalisation and risk of severe disease and mortality.

## Methods

This systematic review is the second one of a project registered on PROSPERO (CRD42021247326), carried out according to the Preferred Reporting Items for Systematic Reviews and Meta-Analyses (PRISMA) (Liberati, 2009). The first review focused on the risk of SARS-CoV-2 infection by MEMs compared to the general population of the EU WHO region (Jaljaa, 2022).

The following databases were searched: Medline, Embase, SciSearch, BIOSIS, and ESBIOBASE. PubMed database (which includes medRxiv, bioRxiv, ChemRxiv, arXiv, Research Square, and SSRN) was also searched for preprints.

After receiving the instructions of the working group, the documentation centre developed a preliminary pilot search aimed at balancing recall (sensitivity) and precision (specificity), using the following keywords: migrants, ethnic minorities, mortality, hospitalisation, complications, intensive care unit, SARS-CoV-2, and Covid-19. Later on, a tailored search strategy was performed, including both Medline and Embase subject headings (i.e., Mesh and Emtree terms, respectively) as well as free-text words related to the subject of the review. The complete search strategy is available as supplementary material. The last database search was carried out on the 19th of March 2021. During the query session, duplicate citations due to databases overlap were removed and the results were exported to Microsoft Excel to facilitate further data analysis and management. Additional duplicates, not automatically intercepted, were manually removed based on a review of the titles.

### Inclusion and Exclusion Criteria

We included studies reporting differential impact/clinical consequences of Covid-19 on MEMs compared to the general population, in the 53 countries belonging to the WHO European Region. International studies reporting data on WHO European Countries were also included. In particular, eligible studies reported quantitative data on outcomes of Covid-19, including negative health consequences, hospitalisation, access to intensive care unit (ICU) and mortality due to SARS-CoV-2 infection. For the definition of ‘migrant’, we referred to the International Organisation for Migration (IOM) glossary (IOM, 2019; sAppendix 3). We sourced the definition of ‘refugee’ from the convention and protocol relating to the status of refugees of the United Nations High Commissioner for Refugees (UNHCR, 2010; sAppendix 3). At last, we defined ‘ethnic minorities’ according to the European Centre for Disease Prevention and Control (ECDC, 2022; sAppendix 3). However, it must be noted that there is no universally accepted definition of such terms and that the differences between migrants and ethnic minorities are nuanced and dependent on the country context.

We collected studies published from January 1, 2020, to March 19, 2021, in English, Italian, French and Spanish. In our review, we included primary and secondary quantitative and quali-quantitative studies (cross-sectional, case-control, cohort, intervention, case-series, prevalence or ecological studies); purely qualitative studies were excluded. Regarding the publication type, comments, opinions, editorials and news were excluded; letters were included if containing original quantitative data. The selected reviews were excluded if their primary studies had already been taken into consideration. Nine researchers, appropriately trained and constantly monitored, were involved in the study selection and assessment and in data extraction. Two at a time screened, independently, title and abstract against eligibility criteria and, later, assessed eligibility for inclusion by reading the full texts. Any disagreements were resolved by discussion between the two reviewers. If it was not possible, an assessment group intervened to solve the disagreement.

### Critical Appraisal, Data Extraction and Synthesis

The study quality was assessed independently by two researchers, using the appropriate Joanna Briggs Institute Critical Appraisal Tools (Critical Appraisal, 2022) for each study design. Quality scores were calculated as the number of positive answers out of the number of applicable questions and converted into percentages. Studies with a score of 80–100% were considered ‘high quality’, 60–79% ‘medium quality’ and 0–59% ‘low quality’. Low-quality studies were not excluded but contributed to the final synthesis.

Data were sought for the following outcomes: hospital admission, severe Covid-19 and ICU admission and mortality*.* The extraction of relevant information from the included documents was carried out by one researcher, using an appropriate extraction form. Then, another researcher checked the information collected. The items extracted included the following: bibliographic reference, publication country, language, type of study, study period, objectives, population, comparison population if available, diagnostic methods, observation setting, outcomes, results, conclusions, limits, comments and study quality.

Disagreements both in quality assessment and in data extraction were resolved by discussion between the two researchers. If it was not possible, an assessment group stepped in for a final decision.

Data of the included studies were narratively described and gathered according to the three main outcomes (hospital admission, severe Covid-19 and ICU admission and mortality). In Table 1, the following dimensions were collected: study period, study design, study population, sample size, main outcomes and their measures of effect (in terms of incidence, prevalence, morbidity rates, rate ratios, odds ratios, relative risks and hazard ratios); the study quality in terms of risk of bias was also included in the table.

**Table 1:** Summary of the studies retrieved by the systematic search of the databases

Citation	Period	Study design	Population (subgroups)	Sample size	Outcomes			Effect measures	Study quality
					M	Ha	ICUa/SO		
Alaa, 2020 (UK)	Feb–Apr 2020	Retrospective cohort study	Ethnic minorities	6,068	x			HR	Medium
Aldridge, 2020 (UK)	Mar–Apr 2020	Cross-sectional study	Ethnic minorities	16,272	x			SMR	High
Apea, 2021 (UK)	Jun–May 2021	Cohort study	Ethnic minorities	1,737	x		x	Survival, HR	High
Atkins, 2020 (UK)	Mar–Apr 2020	Cohort study	Ethnic minorities (hospital inpatients)	269,07	x	x		OR	Medium
Aw, 2020 (UK)	Mar–Apr 2020	Cohort study	Ethnic minorities (aged ≥65)	677	x			HR	High
Ayoubkhani, 2021 (UK)	Mar–May 2020	Retrospective cohort study	Ethnic minorities (> 9 years old)	47,872,412	x			OR	High
Bannaga, 2020 (UK)	Jan–Apr 2020	Retrospective cohort study	Ethnic minorities	321	x		x	OR, RR	Medium
Baronio, 2021 (UK)	Feb–July 2020	Cross-sectional study	Ethnic minorities	111		x		OR	High
Barron, 2020 (UK)	Apr–May 2020	Population cohort study	Ethnic minorities (patients with or without diabetes)	61,414,470	x			SMR, OR	High
Batty, Deary et al., 2021 (UK)	Mar–Sept 2020	Cohort study	Ethnic minorities	448,664	x			OR	Low
Batty, Gaye et al., 2021 (UK)	Apr–Sept 2020	Cohort study	Ethnic minorities	502,655	x			HR	High
Baumer, 2020 (UK)	Mar–May 2020	Cross-sectional study	Ethnic minorities	52	x		x	RR	High
Bray, 2020 (UK)	Mar–Apr 2020	Ecological study	Ethnic minorities	na	x			OR	High
Canevelli, 2020 (IT)	Feb–Apr 2020	Cross sectional study	Migrants (residents)	2,687	x		x	RR	High
Cheng, 2021 (UK)	Mar–June 2020	Cohort study (registry based)	Ethnic minorities	1,781	x			HR	High
Clift, 2020 (UK)	Jan–Apr 2020	Cohort study	Ethnic minorities	6,083,102	x	x		HR	High
Clough, 2021 (UK)	Mar–Apr2020	Cross-sectional study	Ethnic minorities	12,805	x			OR	High
Corcillo, 2021 (UK)	Mar–Apr 2020	Cohort study	Ethnic minorities (diabetes-related end stage renal disease patients)	39		x		OR	High
Daras, 2021 (UK)	Mar–May 2020	Ecological study	Ethnic minorities	na	x			Beta coefficients (linear regression models)	High
Davies, 2020 (UK)	Apr–May 2020	Retrospective cohort study	Ethnic minorities (<18 years old)	78			x	Proportion	High
Davies, 2021 (UK)	Sept 2020–Feb2021	Cohort study	Ethnic minorities	2,245,263	x			Survival analysis, prevalence, crude overall mortality rates, HRs	High
de Lusignan, 2020 (UK)	Jan–May 2020	Cross-sectional study	Ethnic minorities	4,413,734	x			OR	High
Dennis, 2021 (UK)	Mar–June 2020	Retrospective cohort study	Ethnic minorities	21,082	x			HR	High
Dennis, 2021 (UK)	Mar–July 2020	Cohort study	Ethnic minorities (patients with type II diabetes in HDU or ICU)	19,256	x		x	Rate	High
Di Girolamo, 2020 (IT)	Mar–Apr 2020	Ecological study	Migrants	13,418	x			SMR, RR	High
Dite, 2021 (UK)	To Sept 2020	Population-based case-control study	Ethnic minorities	1,582			x	OR	High
Drefahl, 2020 (SE)	Mar–May 2020	Population-based cohort study	Migrants	7,775,054 pers/years	x			OR	High
Elliott, 2021 (UK)	Jan–Sept 2020	Cohort study	Ethnic minorities	473,55	x			Incidence rates	High
Etienne, 2020 (FR)	Mar–Apr 2020	Cohort study	Ethnic minorities (HIV+)	54			x	Probability	High
Fabiani, 2021 (IT)	Feb–July 2020	Cross-sectional study	Migrants	213,18	x	x	x	RR	High
Farrell, 2021 (IRE)	Mar–May 2020	Cohort study	Ethnic minorities	257	x		x	Adjusted HR	High
Felsenstein, 2020 (UK)	Mar–June 2020	Cohort study	Ethnic minorities (paediatric patients)	29		x	x	Proportion	Medium
Ferrando-Vivas, 2021 (UK)	Mar–June 2020	Cohort study	Ethnic minorities	9,99	x			HR	Medium
Galloway, 2020 (UK)	Mar–Apr 2020	Cohort study	Ethnic minorities	1,173	x		x	HR	High
Giorgi Rossi, 2020 (IT)	Feb–Apr 2020	Population-based cohort study	Migrants	2,653	x	x		RR, HR	High
Gopal Rao, 2021 (UK)	Mar–Apr 2020	Cross-sectional study	Ethnic minorities	4981	x		x	OR	High
Guijarro, 2021 (ESP)	Feb–Apr 2020	Population-based cohort study	Migrants	1,036	x		x	Proportion	High
Harris, 2020 (UK)	Mar–Apr 2020	Ecological study	Ethnic minorities and migrants	na	x			SMR	High
Harrison, 2020 (UK)	Feb–May 2020	Cohort study	Ethnic minorities	30,693	x		x	HR	Medium
Hippisley-Cox, 2020 (UK)	Up to Apr 2020	Retrospective cohort study	Ethnic minorities	19,486			x	HR	Medium
Holman, 2020 (UK)	Feb–May 2020	Cohort study (registry based)	Ethnic minorities	3,138,410	x			HR	High
Indseth, 2021 (NO)	Mar–Oct 2020	Register-based surveillance	Migrants	16,232		x		OR	High
Joy, 2020 (UK)	Jan–May 2020	Cross-sectional study	Ethnic minorities	56,628	x			OR	High
Kakkar, 2020 (UK)	Mar–Apr 2020	Cohort study (registry based)	Ethnic minorities	3018		x		OR	Low
Ken-Dror, 2020 (UK)	Mar–Apr 2020	Cohort study	Ethnic minorities	407	x			RR, OR	High
Kolhe, 2020 (UK)	Mar–May 2020	Retrospective cohort study	Ethnic minorities	4,535	x		x	OR	High
Kuo, 2021 (UK)	Mar–Apr 2020	Cohort study	Ethnic minorities	339,285	x			SAR, SMR, HR	High
Lassale, 2020 (UK)	Mar–Apr 2020	Cohort study	Ethnic minorities	340,966		x		OR	High
Livingston, 2020 (UK)	Mar–Apr 2020	Cross-sectional study	Ethnic minorities (age ≥65 or with dementia)	131	x			Prevalence, OR	High
Lo, 2020 (UK, USA)	Mar–May 2020	Cohort study	Ethnic minorities	2,414,601 (US 179,873; UK 2,234,728)		x		Adjusted OR	Medium
Lok, 2020 (UK)	Mar–Apr 2020	Cohort study (registry based)	Ethnic minorities	343			x	Proportion	Medium
Mahil, 2021 (Int)	Mar–July 2020	Cohort study (registry based)	Ethnic minorities (patients with psoriasis)	1,626		x		OR	High
Miles, 2020 (UK)	Up to May 2020	Case-control study	Ethnic minorities	377	x			HR	High
Moody, 2021 (UK)	Mar–May 2020	Cohort study	Ethnic minorities (aged ≥18)	164	x			Proportion	Medium
Navaratnam, 2021 (UK)	Mar–May 2020	Observational retrospective study	Ethnic minorities (aged ≤18)	91,541	x			OR, RR	High
Nazroo, 2020 (UK)	Apr–May 2020	Ecological study	Ethnic minorities	na	x			SMR, HR	High
Nikoloudis, 2020 (UK)	Mar–Apr 2020	Cross-sectional study	Ethnic minorities	na	x			SMR	High
Norman, 2021 (ESP)	Mar–May 2020	Cross-sectional study	Ethnic minorities	2,345	x		x	OR	High
Papageorgiou, 2020 (UK)	Mar–2020	Cohort study	Ethnic minorities	613	x		x	OR, RR, median, IQR	Medium
Patel A, 2020 (UK)	Mar–Apr 2020	Retrospective cohort study	Ethnic minorities (hospitalised population)	645	x		x	RR, OR	High
Patel AP, 2020 (UK)	Mar–Apr 2020	Cohort study	Ethnic minorities	418,794		x		OR	High
Perez-Guzman 2021, (UK)	Feb–May 2020	Retrospective cohort study	Ethnic minorities	614	x			*Z* scores	High
Perkin, 2020 (UK)	Mar–Apr 2020	Death case series	Ethnic minorities	573	x			OR	High
Platt, 2020 (UK)	Up to May 2020	Ecological study	Ethnic minorities	na	x			Rate, RR	Medium
Richards-Belle, 2020 (UK)	Feb–Aug 2020	Cohort study	Ethnic minorities	10,834			x	Proportion	Medium
Rose, 2020 (UK)	Up to Apr 2020	Ecological study	Ethnic minorities	147 upper-tier local authorities of around 370,000 people each	x			RR	High
Rostila, 2021 (SE)	Jan–May 2020	Population cohort study	Migrants (aged 21+ who have lived in Sweden for at least 2 years)	1,778,670	x			RRs, regression model, sensitivity analyses	High
Russell, 2020 (UK)	Feb–May 2020	Cross-sectional study	Ethnic minorities	156	x		x	OR, HR	High
Saban and Wilf-Miron, 2020 (IL)	Feb–June 2020	Cohort study	Ethnic minorities	23,345	x			RR, SMR	Low
Santorelli, 2020 (UK)	Mar–Apr 2020	Cohort study	Ethnic minorities	1,276	x			RR	High
Sapey, 2020 (UK)	Mar–Apr 2020	Cohort study	Ethnic minorities	2,217	x	x		SAR, SMR, HR	High
Sattar, 2020 (UK)	Mar–May 2020	Cohort study	Ethnic minorities	374,503	x			RRs, *p*-interaction	High
Savino, 2020 (UK)	Mar–June 2020	Retrospective cohort study	Ethnic minorities (adults with kidney failure)	2,385	x			HR	High
Singh, 2021 (UK)	Mar–May 2020	Cohort study (registry based)	Ethnic minorities	228,632	x	x		OR	Medium
Staines, 2021 (UK)	Mar–May 2020	Cohort study	Ethnic minorities	177	x		x	Mean, *p* value	Medium
Svensson, 2021 (SE)	Mar–May 2020	Case-control study	Migrants	11,946			x	OR	High
Swann, 2020 (UK)	Jan–July 2020	Multicentre cohort study	Ethnic minorities (<19 years old)	651			x	OR	Medium
Thompson, 2020 (UK)	Mar–May 2020	Retrospective cohort study	Ethnic minorities	470	x			OR	High
Thomson, 2020 (UK)	Mar–June 2020	Cohort study	Ethnic minorities	156	x			OR	High
Verma, 2021 (UK)	na	Cohort study	Ethnic minorities (consultant physicians)	13,5	x			HR	Medium
Williamson, 2020 (UK)	Feb–May 2020	Cohort study (registry based)	Ethnic minorities	17,278,392	x			HR	High
Zakeri, 2020 (UK)	Mar–June 2020	Cohort study and case-control study	Ethnic minorities (aged ≥18)	5,315	x	x		HR, OR	High

Abbreviations: *ESP*, Spain; *FR*, France; *Ha*, hospital admission; *HR*, hazard ratio; *ICUa/SO*, intensive care unit admission/severe outcome; *Int*, international; *IQR*, interquartile range; *IL*, Israel; *IRE*, Ireland; *IT*, Italy; *M*, mortality; *NO*, Norway; *OR*, odds ratio; *RR*, relative risk; *SAR*, standardised admission ratio; *SE*, Sweden; *SMR*, standardised mortality ratio; *UK*, United Kingdom; *USA*, United States of America

## Results

### Literature Search and Selection

**Fig. 1:**
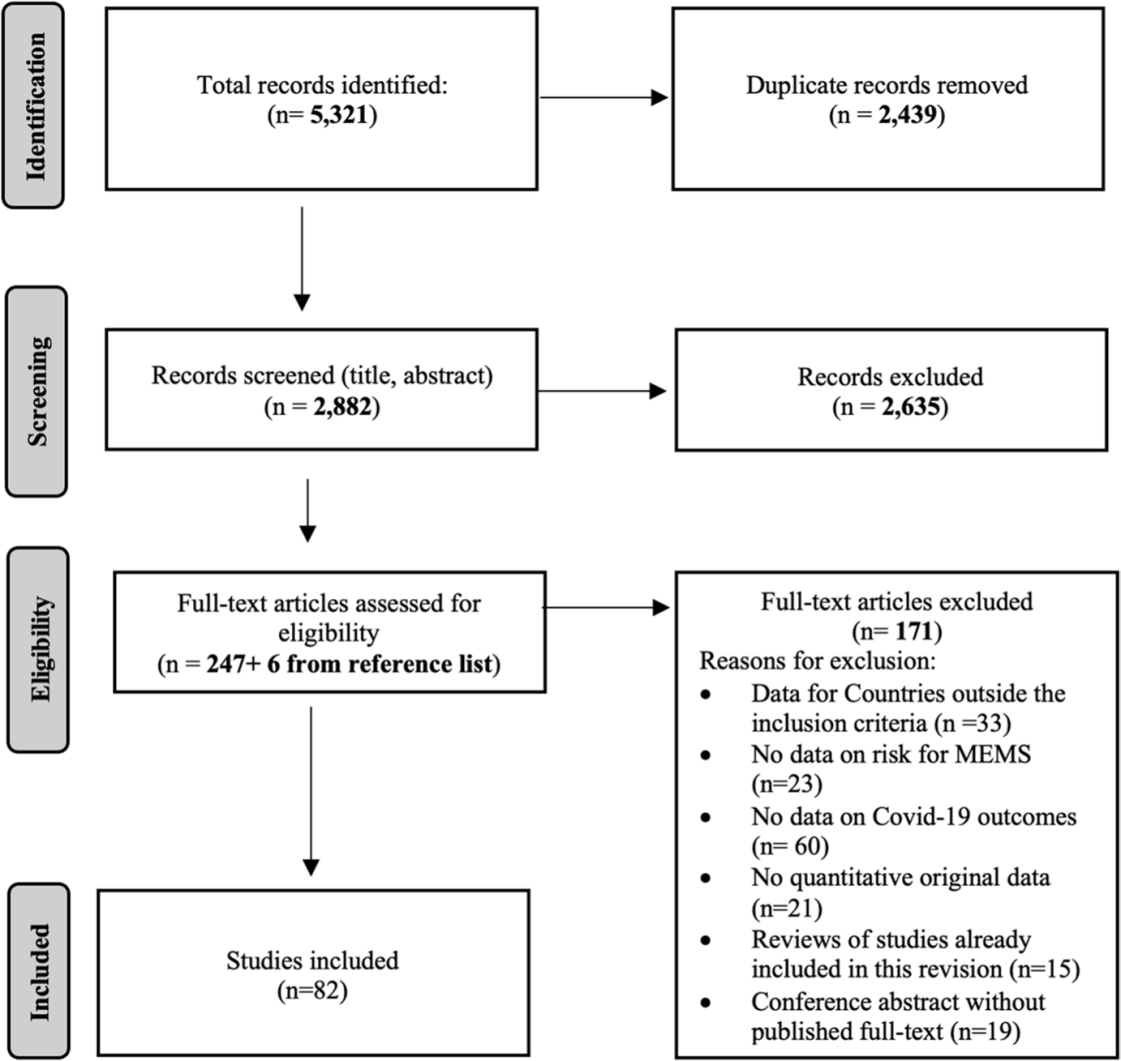
PRISMA flow diagram

The systematic search of the literature concerning the study question identified 5,321 records on databases; after the removal of 2,439 duplicates (2,045 automatically intercepted and 394 by title review), 2,882 records were screened by title and abstract. Two hundred fifty-three were found eligible for full text reading, and 6 more studies were added from the reference lists of the eligible studies. From the resulting 253 studies, 82 records met all the inclusion criteria and were analysed for a quality appraisal. Figure 1 shows the flow diagram of identified studies.

Sixty-two studies were considered ‘high quality’, 17 ‘medium quality’ and only three studies (3.6%) had a ‘low quality’ level. Table 1 shows the characteristics of the included studies.

No studies specifically concerning refugees were found. The studies investigating the migrant population compared to the native one, referred to migrants as persons born outside the country in object.

The findings have been reported according to the outcome taken into consideration as follows: hospital admission, severe Covid-19 and ICU admission and mortality.

### Hospital Admission

We found 16 sources reporting the hospital admission rate and risk for Covid-19 among MEMs, including 10 from the UK, 1 from Norway, 3 from Italy and 2 international studies. Two of these regarded the paediatric population, 2 specific groups of patients with comorbidities and 12 the general adult population.

Fifteen studies out of 16 reported that MEMs had a higher risk of hospital admission for Covid-19 compared to native/White ethnicity. The hospitalisation rate varied markedly depending on ethnicity and country of birth. A cohort study from the UK (Sapey, 2020) reported a standardised admission ratio (SAR) for South Asian women 74% higher (SAR 1.7, 95% CI [1.5–2.0]) and for South Asian men 63% higher (SAR 1.6, 95% CI [1.4–1.9]) than the standard population (2011 census data by age and sex), while admission rates were similar to the expected rates of the standard population for Black ethnicity. Conversely, a second cohort study from the UK (Singh, 2021) found that the South Asian ethnic group was less likely to be hospitalised for Covid-19 compared with the White ethnic reference category (adjusted OR 0.56, 95% CI [0.44 to 0.70]), whereas the Black ethnic group had an increased risk (adjusted OR 2.08, 95% CI [1.70 to 2.57]). These findings were partially confirmed by two cohort studies (Atkins, 2020; Zakeri, 2020), where only Black ethnicity was associated with higher odds of admission (adjusted OR 2.85, 95% CI [1.71–4.74] and 3.1, 95% CI [2.6–3.7], respectively), whereas no increase in admission risk was associated with Asian ethnicity.

Two studies, that used the UK Biobank cohort as data source, reported that both Black (OR 3.1, 95% CI [2.0–4.8] [30] and 4.32, 95% CI [3.00–6.23] [31]) and Asian participants (OR 2.0, 95%CI [1.2–3.1] and 2.12, 95% CI [1.37–3.28]) were at increased risk of hospitalisation when compared to White participants.

A further cohort study (Clift, 2020), that used the UK Qresearch database, reported a higher adjusted hazard ratio (HR) of hospital admission for Covid-19 in the BAME (Black, Asian and minority ethnic) population compared to the White population, especially for the Caribbean (adjusted HR women 2.01, 95% CI [1.71–2.35] and men 2.29, 95% CI [1.99–2.63]) and the Black population (adjusted HR women 2.30, 95% CI [1.97–2.68] and men 2.59, 95% CI [2.27–2.97]).

These findings were also confirmed by an international cohort study (Lo, 2020): in the UK, the adjusted OR for Covid-19 requiring a hospital visit was elevated across ethnic minorities compared to non-Hispanic White participants (adjusted OR 2.11, 95% CI [1.81–2.46]).

Among the studies that described the immigrant population, a Norwegian register-based surveillance (Indseth, 2021) reported a higher number of hospitalisations relative to notified cases of Covid-19 in immigrants compared to non-immigrants, with an increased rate of hospitalisation in many immigrant groups when adjusted for age and sex, ranging from 7/100.000 (Polish-born immigrant group) to 354/100.000 (Somalian-born group) depending on the specific immigrant community, compared with the non-immigrant population that had a hospitalisation rate of 21 per 100.000.

In two Italian studies considering the adult population (Fabiani, 2021; Giorgi Rossi, 2020), immigration status was found to be associated with a higher risk of hospitalisation (adjusted RR 1.39, 95% CI [1.33–1.34] and HR 1.3, 95% CI [0.99 to 1.81], respectively), especially among non-Italian nationals from low Human Development Index (HDI) countries (adjusted RR 1.59, 95% CI [1.48–1.71]) (Fabiani, 2021).

#### Paediatric Population

The first of the two papers regarding the paediatric population (Felsenstein, 2020) found that, among 29 children admitted to tertiary paediatric centres in the English North West with a diagnosis of PIMS-TS (Paediatric Inflammatory Multisystem Syndrome Temporally associated with SARS-CoV-2), children of BAME background were overrepresented when compared to the composition of the general population in the region*.* The second one (Baronio, 2021) reported that among 111 children, the ratio of hospitalised foreign patients was significantly higher than expected when compared with the native Italian patients (OR 1.76, 95% CI [1.16–2.66]). Children from Africa had significant higher odds of being hospitalised with SARS-CoV-2 infection than Italian children (OR = 2.76, 95% CI [1.56–4.87]) and than all other foreign ethnicities combined (OR 2.03, 95% CI [1.00–4.13]).

#### Patients with Comorbidities

Among the studies that analysed patients with comorbidities, a study on a cohort of 39 patients with diabetes-related end stage renal disease (Corcillo, 2021) observed a high prevalence of patients of Afro-Caribbean ethnicity hospitalised with Covid-19 with a 73% and 54% prevalence in renal transplant and haemodialysis (HD) groups, respectively. This contrasted markedly with the much lower prevalence of Afro-Caribbean people in the renal transplant population attending the hospital for routine care (18% of renal transplant and 42% of HD patients of Afro-Caribbean origin).

Also, an international study (Mahil, 2021) on a total of 374 patients with psoriasis and confirmed or suspected Covid-19 from 25 countries stated that significant associations with increased hospitalisation rate were observed for non-White ethnicity (adjusted OR 3.15, 95% CI [1.24–8.03]) compared with the White one.

Only one UK study (Kakkar, 2020), on a cohort of 3,018 adult patients tested for Covid-19 at Sheffield Teaching Hospital, found no significant difference between BAME and White groups in terms of overall admissions (*p*=0.083).

### Severe Covid-19 and ICU Admission

We retrieved 26 studies investigating severe consequences of Covid-19 and ICU admission rates among MEM population. Nineteen studies were conducted in the UK, two in Spain, two in Italy, and one in Ireland, Sweden and France, respectively.

Some studies conducted in the UK reported a higher ICU admission risk for Asian and Black (Lok, 2020) or overall BAME population (Bannaga, 2020; Baumer, 2020; Dennis, 2021; Galloway, 2020; Gopal-Rao, 2021; Harrison, 2020; Hippisley-Cox, 2020; Richards-Belle, 2020)*.* On the other hand, among patients admitted to ICU with other viral pneumonias, the proportion of non-White patients was smaller (11.2% versus 32.6%) (Richards-Belle, 2020).

Regarding presenting symptoms and the need for invasive treatments, in a multicentre cohort study (Papageorgiu, 2020), Asian patients presented more frequently with deep vein thrombosis and/or pulmonary embolism (adjusted OR 4.10, 95% CI [1.49–11.27]) and had a higher rate of mechanical ventilation (*p*=0.007). Afro-Caribbeans required more frequently urgent renal replacement therapy (adjusted OR 2.11, 95% CI [1.15–3.86]), had a higher risk of cardiovascular consequences and resorted more often to ECMO (extracorporeal membrane oxygenation) (*p*=0.014). According to Apea et al. (Apea, 2021), both Asian and Black patients were more likely to receive invasive ventilation (OR 1.54, 95% CI [1.06–2.23] and OR 1.80, 95% CI [1.20–2.71], respectively). Dite et al. (Dite, 2021) found a 43% increased risk for severe Covid-19 among non-Whites (adjusted OR 1.43, 95% CI [0.99–2.05]), even though with marginal significance (*p*=0.06). Conversely, Harrison et al. (Harrison, 2020) found no significant differences in the distribution of severity scores at hospital admission between ethnic groups. However, abnormal liver function during hospitalisation for Covid-19 (Lok, 2020) was more common among Asian patients compared with White ones (59.0% vs 39.8%, *p*=0.026). Being from sub-Saharan African origin was independently associated with severe and critical forms of Covid-19 in patients living with HIV (Etienne, 2020). Compared with the French whole out-patient population, the proportion of Covid-19 patients in those from sub-Saharan African origin was higher (44.6 vs 29.7%) and these patients counted for 31.4% of those with moderate, 57.1% with severe and 100% with critical Covid-19 (*p*=0.08). According to a Swedish study (Svensson, 2021), among patients with a region of birth outside the EU15^1^, diabetes had a stronger association with severe Covid-19 compared with patients born within EU15 (*p*=0.004). An Italian study (Fabiani, 2021) reported a higher ICU admission rate in non-Italian compared to Italian nationals (adjusted RR 1.19, 95% CI [1.07–1.32]), particularly in those from medium and low HDI countries (adjusted RR 1.25, 95% CI [1.11–1.42] and adjusted RR 1.16, 95% CI [0.93–1.44], respectively). A study conducted in Spain (Norman, 2021) underlines that ICU admission was more frequent in non-Europeans (OR 1.43, 95% CI [1.03–1.98]). Also, in another study from Ireland (Farrell, 2021), compared with White Irish patients, all other ethnic groups, including other White and BAME, had an approximately fourfold increased risk of ICU admittance after adjusting for age (adjusted HR=4.22, 95% CI [1.45–12.31] and 4.58, 95% CI [1.33, 15.84], respectively).

### Paediatric Population

Two articles (Davies, 2020; Felsenstein, 2020) analysed cohorts of children under 18 years of age with PIMS-TS. Asian and Afro-Caribbean patients were over-represented (28% Asian and 47% Afro-Caribbean), despite accounting for 7% and 8% of the UK population, respectively. Out of 78 PIMS-TS children, 61 (78%) were of ethnic minorities (Davies, 2020). They were also more likely to present with clinical signs of hypoperfusion or shock (*p*=0.04) and abnormalities on echocardiogram (*p*=0.02) (Felsenstein, 2020). Moreover, BAME children were at higher risk of PIMS-TS (*p*=0.004), and children with this syndrome showed higher ICU admission rates (*p*<0.001) (Swann, 2020).

Six papers did not report any increased risk for people from ethnic minorities. Russell et al. (Russel, 2020) found no increased risk of severe Covid-19 in ethnic minorities compared to White patients, and Patel et al. (Patel A, 2020) and Staines et al. (Steines, 2021) delineated no difference for ICU admission risks. A study conducted in Spain (Guijarro, 2021) reported the unadjusted rate of ICU admission as being higher for Spaniards (63%) compared to migrants (33%). Canevelli et al. (Canevelli, 2020) found no significant difference between people with a migration background and native Italian individuals regarding admission to ICU, times to clinical milestones (all *p*>0.05) and proportion of development of ARDS (acute respiratory distress syndrome) (97.0% of natives and 96.9% of migrants). According to a UK study, ethnicity does not appear to be an independent risk factor for developing acute kidney failure (AKI) with Covid-19 either (*p*=0.443) (Kolhe, 2020).

### Mortality

The research retrieved 65 studies investigating the difference in mortality risk between MEMs and non-ethnic-minority populations, of which 43 documented an association between mortality and being MEMs.

Several studies reported an overall increased mortality for BAME population after adjusting for age and sex (Apea, 2021), comorbidities (Alaa, 2020; Papageorgiu, 2020) and socio-economic characteristics (Batty, Deary et al., 2021; Nikoloudis, 2020; Perkin, 2020; Williamson, 2020). Among those observing a higher mortality rate in the BAME population, 9 found the Asian ethnicity to be at increased risk of mortality than other ethnic minorities, compared to the White population. A cohort study and an observational retrospective study from the UK reported that the Asian population had greater odds of death compared to White people (adjusted OR 1.99, 95% CI [1.22–3.25] (Patel A, 2020) and adjusted OR 1.21, 95% CI [1.13–1.30] (Navaratnam, 2021), respectively), compared to other ethnic minorities. This was also confirmed by Ferrando-Vivas (Ferrando-Vivas, 2021) and Cheng (Cheng, 2021), who, in a multivariable Cox proportional hazards model for death within 30 days of start of critical care, observed that patients with an Asian background had an adjusted HR of 1.27, 95% CI [1.15–1.40] and HR 1.40, 95% CI [1.08 to 1.81], respectively. This last result was corroborated by a sensitivity analysis conducted, restricted to PCR-confirmed Covid-19 patients, among which people of Asian ethnicity had higher mortality risk (HR 1.5, 95% CI [1.1 to 2.1]) (Cheng 2021). Other cohort studies from the UK analysed different Asian subgroups of population and found in multivariable models that South Asian ethnicity was associated with a significantly higher risk of death (adjusted HR 1.4, 95% CI [1.2 to 1.8] [26]; adjusted HR 1.19, 95% CI [1.05 to 1.36] (Harrison, 2020); adjusted HR 2.00, 95% CI [1.13–3.51] (Miles, 2020), respectively), compared to the White group. In other studies, the Black population was found to be at increased risk of death, with adjusted OR ranging from 1.17, 95% CI [1.03–1.33], to 3.44, 95% CI [1.48–8.00] (Atkins, 2020; de Lusignan, 2020; Elliott, 2021; Joy, 2020; Kuo, 2021; Perez-Guzman, 2021; Singh, 2021). Although Singh et al. (Singh, 2021) pointed out the non-significant association between in-hospital Covid-19 case fatality and ethnicity in general, the Black population showed higher absolute and adjusted mortality occurred in the Black population compared to White people and other ethnic minorities. Batty et al. (Batty, Gaye et al., 2021) reported a higher mortality risk adjusted for sex and age for both Black and Asian patients (age and sex adjusted OR 7.25; 4.65, 11.33 and 1.98; and 1.02 and 3.84, respectively). After a separate adjustment for lifestyle, socio-economic factors and comorbidities, the association resulted significant only in the Black population (adjusted OR 4.29, 95% CI [2.67–6.88]). Moreover, the risks across ethnic groups were analysed in detail. In a cross-sectional study conducted in the UK (Clough, 2021), Bangladeshi/Pakistani, Black and Indian ethnic groups (for both sexes) had increased adjusted odds of dying from Covid-19 when compared with a baseline White group. These findings were confirmed by Aldridge (2020) and Platt (2020), who reported an increased risk of death for Black Africans, Pakistanis, Black Caribbeans, Indians and Bangladeshi ranging from 1.7 to 4.1. In a cross-sectional study on 52 patients admitted to ICU in the Royal Gwent Hospital (Baumer, 2020), people of BAME minority ethnic descent represented 35% of deaths (6 of 17) and 72% (13 of 18) of BAME patients were found to reside in geographical areas representing the 20% most deprived in Wales. Findings of an ecological study confirmed that areas with higher percentages of Black and Asian (except Chinese) ethnic groups had higher mortality rates (Harris, 2020). According to Daras et al. (Daras, 2021), each standard deviation increase in the proportion of BAME population was associated with a 8% increase in the Covid-19 mortality rate (IRR 1.08, 95% CI 1.03 to 1.13). Likewise, each 1% increase in the proportion of BAME population was associated with a 1% increase in the Covid-19 mortality rate (IRR 1.01, 95% CI 1.01 to 1.02) (Rose, 2020) or an increase in Covid-19-related deaths by 5.12 (95% CI [4.00 to 6.24]), per million (Nazroo, 2020). Moreover, a negative association between the proportion of White people and rate of death related to Covid-19 was found (*r*=−0.6) (Bray, 2020). A fully adjusted model (Ayoubkhani, 2021) described the mortality HRs as higher for Black females (HR 1.29, 95% CI [1.18–1.42] and for Black (HR 1.76, 95% CI [1.63–1.90], Bangladeshi/Pakistani (HR 1.35, 95% CI [1.21 to 1.49] and Indian (HR 1.30, 95% CI [1.19 to 1.43]) males compared to the White counterparts (Ayoubkhani, 2021). In a UK cohort study of 13,500 consultant physicians aged 50–59 years (Verma, 2021), the authors highlighted the striking effects of ethnicity on the risk of Covid-19-related death for both BAME men (ranging from HR 1.59 to 2.35) and women (ranging from HR 1.33 to 1.48) compared to the White population. Similar observations were reported by another UK cohort study (Clift, 2020), according to which men and women of BAME background registered higher risks of Covid-19-related death compared to White counterparts. However, men of each ethnic group taken into consideration had a greater HR of death than women (women of Indian 1.89, 95% CI [1.43–2.51], Caribbean 1.68, 95% CI [1.29–2.20], Black African 1.98, 95% CI [1.39–2.83], and other ethnic groups 1.73, 95% CI [1.28–2.35] compared to white women; men of Indian 1.59, 95% CI [1.25–2.01], Pakistani 1.84, 95% CI [1.39–2.44], Bangladeshi 2.27, 95% CI [1.65–3.12], other Asian 2.02, 95% CI [1.49–2.74], Caribbean 2.06, 95% CI [1.6–2.57], Black African 3.03, 95% CI [2.42–3.80], Chinese 2.47, 95% CI [1.49–4.09] and other ethnic groups 2.04, 95% CI [1.60–2.58] compared to White men).

The research retrieved only four studies conducted outside the UK reporting an increased mortality risk for migrants compared to the native population, two from Sweden and two from Italy. Drefahl et al. (Drefahl, 2020) observed that immigrants from low- and middle-income countries (LIC and MIC) of the Middle East and Northern Africa displayed more than three times higher mortality among men (HR 3.13, 95% CI [2.51–3.90]) and two times higher among women (HR 2.09, 95% CI [1.52–2.89]), whereas immigrants from other LIC and MIC experienced a mortality risk more than doubled among men (HR 2.20, 95% CI [1.81–2.69]) and almost a 1.5 times higher among women (HR 1.45, 95% CI [1.12–1.90]), compared to those born in Sweden. Male immigrants from high-income countries displayed 19% higher mortality (HR 1.19, 95% CI [1.01–1.39]). Also, an Italian cross-sectional study (Fabiani, 2021) observed a higher fatality rate between non-Italian nationals from low HDI countries compared to Italian nationals (adjusted RR 1.32, 95% CI [1.01–1.75]). In the other Swedish study (Rostila, 2021), in a sample of people aged more than 21 years, the authors reported higher mortality rate ratios among migrants from the Middle East (adjusted RR 1.96, 95% CI [1.56–2.46]), Africa (adjusted RR 1.70, 95% CI [1.17–2.47]) and non-Sweden Nordic countries (adjusted RR 1.25, 95% CI [1.03–1.52]) than persons born in Sweden. These findings were confirmed by the sensitivity analysis.

An Italian ecological study (Di Girolamo, 2020) delineates a positive monotonic gradient for Covid-19 mortality by the percentage of foreign resident population, considered a proxy for social and economic disadvantage. All the ecological measures were grouped in population quintiles of the regional distribution. Mortality rate ratios (MRRs) were found to be significantly increased in the fourth and fifth quintiles for men (Q4: 1.34, 95% CI [1.16–1.54]; Q5: 1.57, 95% CI [1.37–1.80]) and in the fifth one for women (Q5: 1.20, 95% CI [1.02–1.42]), compared to the respective first quintile.

#### Patients with Comorbidities

In a population of cancer patients (Russell, 2020) being of Asian ethnicity had a positive association with risk of Covid-19 death as compared to White (HR 3.73, 95% CI [1.28–10.91]) and in a retrospective cohort study on patients in haemodialysis (Savino, 2020), Asian patients with Covid-19 were found to have a ‘borderline’ 16% higher mortality risk (HR 1.16 CI 0.94–1.44) than White patients. A strong significant interaction (*p*-interaction=0.002) was also found between the body mass index and non-Whites in increasing the risk of death for Covid-19 (Sattar, 2020). Concerning patients with a diagnosis of both type 1 and 2 diabetes, Covid-19-related mortality was found to be significantly higher in people of Black and Asian ethnicities than in those of White ethnicity (type 1 diabetes: adjusted HR 1.77, 95% CI [1.25–2.49] and adjusted HR 1.57, 95% CI [1.16–2.12], respectively, and type 2 diabetes: adjusted HR 1.63, 95% CI [1.51–1.77] and adjusted HR 1.08, 95% CI [1.01–1.15], respectively) (Holman, 2020). Barron et al. (Barron, 2020) reported higher odds for Covid-19-related death for BAME population both with and without diabetes, with a general adjusted OR of 1.35, 95% CI [1.28–1.42] for Asian people, 1.71, 95% CI [1.61–1.82] for Black people, 1.43, 95% CI [1.23–1.67] for mixed and 1.10, 95% CI [1.01–1.20] for other ethnic minorities. However, a study reported no increased mortality for non-White ethnicity compared to White in patients with type 2 diabetes (*p*=0.08) (Dennis, 2021).

Twenty-one studies did not report an increased mortality risk for people with an ethnic minority background (Aw, 2020; Bannaga, 2020; Canevelli, 2020; Davies, 2021; Dennis, 2021; Farrell, 2021; Galloway, 2020; Giorgi-Rossi, 2020; Gopal-Rao, 2021; Guijarro, 2021; Ken-Dror, 2020; Kolhe, 2020; Livingston, 2020; Moody, 2021; Norman, 2021; Santorelli, 2020; Staines, 2021; Saban & Wilf-Miron, 2020; Thompson, 2020; Zakeri, 2020), and although Thomson et al. (Thomson, 2020) found an association in the univariable analysis between Asian ethnicity and death (OR 2.57, 95% CI 1.02–6.57), in the multivariable model, this finding was not significant (Asian OR 2.94, 95% CI 0.94–9.78).

## Discussion

This systematic review aims at investigating the differential risk of Covid-19 adverse outcomes between MEMs compared to the non-migrant population in the countries of the European Region of WHO. As stated in a previous systematic review (Jaljaa, 2022), this geographical restriction was an attempt to limit the heterogeneity of the target population among different countries.

The various outcomes that emerged from the research have been grouped and reported in the current study as follows: hospital admission, admission to ICU and/or severe Covid-19 symptoms and mortality. Most of the 82 studies included reported an increased risk for adverse outcomes for the MEM population.

### Clinical Outcomes

Differences in hospitalisation rates were considered by 16 studies, those on ICU admission and severe outcomes by 26 studies and differences in mortality risks by 65 studies. Among these, 24 studies investigated more than one outcome. Overall, most studies focused on the differences in the mortality risk.

Even if the research highlighted a clear enhanced risk of adverse outcome and mortality due to Covid-19, the consistency among the studies differs, depending on the outcome taken into consideration.

Regarding hospital admission, almost all the papers reported an increased risk of hospitalisation for MEMs, except one (Kakkar, 2020). However, this study lacked adjusted measures and had a ‘low-quality’ rating according to our quality evaluation.

Among the studies considering ICU admission rates and other severe outcomes, 21 out of 26 studies showed an increased risk of ICU admission and 43 studies out of 65 reported an increased risk of mortality for MEM people. Therefore, a consistent enhanced risk of adverse outcomes for MEMs resulted throughout all the variables considered.

The main factors implied in the genesis of health inequalities identified throughout the retrieved literature are defined as the social determinants of health (SDH). It is well recognised by the scientific community that SDH is deeply involved in shaping the health condition of the individual and the communities and social deprivation has been associated with increased risk of SARS-CoV-2 infection and Covid-19 (Morante-García, 2022; Patel JA, 2020). Indeed, MEM people are usually over-represented among key workers, in low income and/or in public-facing jobs, in precarious contracts or self-employed and they live in larger, intergenerational crowded housing (Lassale, 2020). They are also more likely to present lower levels of language proficiency, which constitute a barrier in accessing health information and leads, therefore, to a lower health literacy (Hayward, 2021; WHO, 2022a, 2022b). Moreover, if not legally resident, migrants may be frightened to access official healthcare services due to fear of legal consequences or repatriation (WHO, 2020).

These factors contribute to increasing the risk of infection of SARS-CoV-2 among this population (Jaljaa, 2022) but might also be involved in the worse clinical presentation and the faster progression of Covid-19 disease (Bambra 2020; Fiorini, 2020) among this group. Barriers such as language, administrative, legal, cultural and social and the fear of a restriction of working activities due to isolation/quarantine may have been obstacles to the early access to healthcare services, thus, leading to a delayed diagnosis (Baumer, 2020; Fabiani, 2021). As reported in studies from the UK, it is also possible that, in areas with MEMs population, an asymmetrical access to healthcare or the quality of care itself could heighten the risk of infection, hospitalisation and mortality (Gopal-Rao, 2021). In addition, a disadvantaged socio-economic status, also supported by structural racism, is likewise related to the higher comorbidity rates observed among MEM population, increasing the susceptibility to poor outcomes consequent to Covid-19 infection (Lo, 2020).

However, even if the hospital and ICU admission is higher for the target population, some studies do not register a subsequent increase in mortality rates (Bannaga, 2020; Gopal-Rao, 2021; Papageorgiu, 2020). These findings were attributed to the small sample size (Bannaga, 2020), and the low overall mortality rates reported by the study (Gopal-Rao, 2021), that could not help in highlighting the possible differences. Some authors justified the fact by clarifying that elderly European patients with severe disease were not being considered for ICU at the beginning of the pandemic, or that, since an increase in comorbidities in the non-European group admitted to ICU should probably have led to worse outcomes and a higher mortality, which was not observed; other determinants, such as genetic factors, may also be playing a role in the outcome for these patients (Norman, 2021). Some other authors left the finding without a proper explanation (Dennis, 2021; Farrell, 2021; Galloway, 2020). On the other hand, Patel addressed physiological factors—such as increased comorbidity burden and/or delayed hospital presentation, as precluding factors for MEM group from critical care. Social and cultural factors such as family and support networks may affect healthcare accessibility which may also impact outcomes (Patel A, 2020).

Some authors identify the different biological susceptibilities of distinct ethnic minorities as a key factor in the interpretation of the higher rates of negative consequences of Covid-19 (Davies, 2020; Norman, 2021). However, regardless of the biological susceptibility factors, the variables that influence the degree of control that individuals exert on their own lives can affect their ability to cope with the biological factors. These variables are identified in the socio-economic conditions which characterise health status. Each factor that disrupts the balance in the coping ability influences the frailty of the individual, worsening health conditions and outcomes (Costa, 2020). The interaction between social components and pre-existing medical conditions which causes a worsening in clinical outcomes, as occurred during the current pandemic, relates to the notion of syndemic (Horton, 2020). Interestingly, especially with regard to the mortality risk, wide differences between ethnic groups were highlighted. The reasons behind these findings could be various and heterogeneous, and further qualitative and quantitative studies are needed to better interpret the phenomenon.

Another factor playing a significant role is the different groupage operated by the researchers, where populations with differences in social, biological conditions and migration histories are included in the same broad category, as ‘Asian’, ‘Black’ and a generic ‘other ethnic minorities’.

Just four studies (Baronio, 2021; Davies, 2020; Felsenstein, 2020; Swann, 2020) focused on the paediatric population. For this reason, it is premature to draw any conclusion and we await a larger body of scientific evidence.

Even though the research was performed in the same days as the one investigating the difference in the risk of SARS-CoV-2 infection (Jaljaa, 2022), the total number of articles retrieved was more than three times higher. The reason could be a higher interest in clinical outcomes related to Covid-19 compared to risk of infection or could also be explained by the fact that hospitals were hit by a massive wave of hospitalisation of Covid-19 patients and the healthcare professionals were focused on reducing the mortality rate. Likewise, the scientific production was directed to this issue (De Felice & Polimeni, 2020; Nowakowska, 2020). Indeed, the articles included in the review considered a timeframe between January and October 2020, which corresponds to the first wave of Covid-19 contagions in the European region, when the immunisation through vaccination was not yet available.

However, even though the European Region of WHO includes 53 countries, the research gathered publications from just eight of them and 81.7% of the studies were conducted in the UK. In this country, the National Healthcare Service (NHS) has information and databases which are more structured and accessible compared to other countries, and do not face comparable difficulties of retrieving ethnicity data for public health purposes.

The current policy in force in some of these nations hinders the collection of data on the ethnicity of the individuals (Balestra & Fleischer, 2018). Even if the clear aim of this policy is to protect those groups of people historically victims of prejudices, it also prevents the researchers from obtaining the required information to properly study their conditions and needs. These data turn out to be essential to formulate adequate social and political responses to the issues raised also by the current pandemic.

Nevertheless, the results of this review highlighted the necessity to take into account the socio-economic barriers that MEMs face, in achieving and preserving their health status. These factors have to be considered by policymakers in developing public health prevention plans.

## Strengths and Limitations

This systematic review is one of the few conducted on the topic (Agyemang, 2021; Hayward, 2021). It is the only one focused specifically on the European Region of the WHO and on both migrants and ethnic minorities. Moreover, the time span taken into consideration was longer, up to the first months of 2021, whereas Agyemang et al. included articles published up to September 2020 and Hayward et al. up to November 2020. The results of the three reviews are consistent in terms of the increased risk of severe consequences of Covid-19 among migrants (Hayward 2021) and ethnic minorities (Agyemang 2021).

Furthermore, this is the second part of a three-query research which aims at providing a thorough picture of the impact of the Covid-19 pandemic on MEM population from the aspects of risk of infection, risk of adverse clinical outcomes and the achievement of healthcare services and vaccination. The research group working on this study is composed of different professional figures operating in various fields related to migration and public health, thus bringing the value of a holistic point of view to the subject.

We also recognise some limitations. Most of the studies identified White ethnicity as the population of comparison, although the definition of ‘White ethnicity’ is broad and does not allow one to distinguish the possible migration background within this group.

On the other hand, the definition of migrant and ethnic minority is loose, meaning that the quantity and quality of the composition of the MEM population can be very different from one place to another. This, therefore, challenges the interpretation of the higher risk of negative outcomes due to Covid-19 in this population.

Nevertheless, we believe that this enhanced risk of Covid-19 severe outcomes and mortality in migrant and ethnic minorities compared to the general population in the European WHO region can be attributed to the unequal distribution of the social determinants of health, since MEM population belongs more often to the most socio-economic disadvantaged section of the population.

## Conclusion

The findings of this systematic review highlight the disproportionate impact of Covid-19 on MEM population in terms of clinical consequences.

It raises, therefore, the urgent need for healthcare policies to take into consideration the higher vulnerability of these people to SARS-CoV-2 infection and worse Covid-19 clinical outcomes and to aim at tackling the socio-economic gradient, acknowledged cause of health inequalities.

Hence, we recommend conducting similar studies to be conducted in countries where the number of publications on this issue is still very scarce or inexistent.

## Supplementary information

Supplementary material The online version contains supplementary material available at sAppendix 1 and 2.

## Footnotes

1 The EU15 comprised the following 15 countries: Austria, Belgium, Denmark, Finland, France, Germany, Greece, Ireland, Italy, Luxembourg, Netherlands, Portugal, Spain, Sweden and the UK. Glossary of statistical terms, OECD.
