# Supplementary material for: Risk of Covid-19 Severe Outcomes and Mortality in Migrants and Ethnic Minorities Compared to the General Population in the European WHO Region: a Systematic Review
Source: J Int Migr Integr. 2023 Jan 11:1–31. Online ahead of print. doi: 10.1007/s12134-023-01007-x (PMC9833641; doi:10.1007/s12134-023-01007-x)
Supplement: Supplementary file 1 — The online version contains supplementary material available at sAppendix 1 and 2. [file 12134_2023_1007_MOESM1_ESM.docx]

**Systematic review: COVID-19 and migrants**

**Supplementary file**

**sAppendix 1.: Study question page 2**

**sAppendix 2: Search strategy page 3**

**sAppendix 3: Definitions page 5**

**sAppendix 1. : Study question**

**Are the immigrant population and ethnic minorities more exposed to the risk of COVID-19 severe outcomes or mortality than the indigenous population?**

**Databases**: Medline, Embase, Biosis, Scisearch, Esbiobase

**Linguages**: English, French, Spanish, Italian

**Years:** from 1^st^ January 2020 to 19^th^ March 2021

**N. Records**: 3276

**sAppendix 2: Search strategy**

L1 66030 S "TRANSIENTS AND MIGRANTS"/CT OR "EMIGRANTS AND IMMIGRANTS"+NT OR MIGRANT+NT/CT

L2 308872 S (MIGRANT# OR IMMIGRANT# OR TRANSIENTS OR IMMIGRATION# OR EMIGRATION#)/TI,AB L3 80824 S HUMAN MIGRATION+NT/CT OR MIGRATION+NT/CT

L4 6996 S (HUMAN OR INTERNATIONAL)(W)MIGRATION#/TI,AB

L5 40180 S (REFUGEE# OR INTERNALLY DISPLACED OR FORCIBLY DISPLACED OR INTERNATIONAL PROTECTION SEEKER# OR ASYLUM SEEKER# OR ASYLEE# OR SUBSIDIARY PROTECTION SEEKER# OR HUMANITARIAN PROTECTION SEEKER#)/TI,AB

L6 903 S UNDOCUMENTED IMMIGRANT/CT

L7 762 S (UNDOCUMENTED OR ILLEGAL OR IRREGULAR)(W)(PERSON# OR PEOPLE# OR INDIVIDUAL# OR POPULATION# OR PATIENT#)/TI,AB

L8 367 S (UNDOCUMENTED OR ILLEGAL OR IRREGULAR)(W)(WORKER# OR ALIEN# OR FOREIGNER# OR STRANGER#)/TI,AB

L9 222 S (UNAUTHORIZED OR UNAUTHORISED)(W)(PERSON# OR PEOPLE# OR INDIVIDUAL# OR POPULATION# OR PATIENT#)/TI,AB

L10 15 S (UNAUTHORIZED OR UNAUTHORISED)(W)(WORKER# OR ALIEN# OR FOREIGNER# OR STRANGER#)/TI,AB

L11 162093 S MINORITY GROUPS/CT OR MINORITY GROUP/CT OR ETHNIC GROUPS/CT OR ETHNIC GROUP/CT

L12 432238 S ETHNIC?(A)MINORIT?/TI,AB OR ETHNIC(W)(GROUP# OR COMMUNIT?)/TI,AB OR ETHNICITY/TI,AB

L13 149438 S ETHNICITY/CT

L14 85430 S RACE FACTORS/CT OR RACE/CT

L15 567646 S RACE#/TI,AB OR RACIAL(W)(GROUP# OR COMMUNIT? OR MINORIT?)/TI,AB

L16 1400938 S L1-L15

L17 2394128 S MORTALITY+NT/CT

L18 7459167 S (MORTALIT? OR LETHAL? OR FATAL? OR DEATH#)/TI,AB

L19 2227437 S "SEVERITY OF ILLNESS INDEX"+NT/CT OR DISEASE SEVERITY+NT/CT

L20 11624616 S (SEVERE? OR SEVERITY OR GRAVITY OR CRITICAL? OR SERIOUS?)/TI,AB

L21 2995543 S "SIGNS AND SYMPTOMS"+NT/CT OR CLINICAL FEATURE/CT

L22 5614985 S (SIGN# OR SYMPTOM# OR SYMPTOMATOLOG?)/TI,AB

L23 1286240 S CLINICAL(W)(FEATURE# OR MANIFESTATION# OR CHARACTERISTIC# OR PRESENTATION#)/TI,AB

L24 212827 S CLINICAL(W)(EFFECT# OR CONSEQUENCE# OR ASPECT#)/TI,AB

L25 4908123 S COMPLICATIONS+NT/CT OR COMPLICATION+NT/CT

L26 4421087 S (COMPLICATION# OR SEQUELA# OR SEQUEL# OR DETERIORAT?)/TI,AB

L27 7950 S ASYMPTOMATIC CARRIER/CT OR ASYMPTOMATIC INFECTION/CT

L28 1014837 S (ASYMPTOMATIC? OR NON SYMPTOMATIC OR PRESYMPTOMATIC OR SILENT OR SUBCLINICAL OR PAUCISYMPTOMATIC)/TI,AB

L29 2877346 S TREATMENT OUTCOME+NT/CT OR CLINICAL OUTCOME/CT

L30 7650158 S OUTCOME#/TI,AB

L31 2469161 S PHYSIOPATHOLOGY/CT OR PATHOPHYSIOLOGY/CT

L32 2935672 S (PHYSIOPATHOLOG? OR PATHOPHYSIOLOG? OR DYSFUNCTION?)/TI,AB

L33 55676 S MULTIPLE ORGAN FAILURE/CT

L34 285877 S ORGAN#(3A)(FAILURE# OR INVOLVE? OR INJUR? OR DAMAGE? OR ALTER?)/TI,AB

L35 22786 S MULTIORGAN(3A)(FAILURE# OR INVOLVE? OR INJUR? OR DAMAGE? OR ALTER?)/TI,AB

L36 13428 S (MULTISYSTEM? OR MULTI SYSTEM?)(3A)(FAILURE# OR INVOLVE? OR INJUR? OR DAMAGE? OR ALTER?)/TI,AB

L37 1734536 S FUNCTION?(3A)(IMPAIR? OR ALTER? OR ABNORMAL? OR AFFECT? OR DAMAGE# OR INJUR? OR INVOLVE? OR WORSEN? OR LOSS?)/TI,AB

L38 574484 S RESPIRATION DISORDERS+NT/CT OR BREATHING DISORDER+NT/CT

L39 27025 S (RESPIRAT? OR BREATH?)(W)DISORDER?/TI,AB

L40 542412 S RESPIRATORY TRACT INFECTIONS+NT/CT OR RESPIRATORY TRACT INFECTION/CT

L41 408320 S RESPIRATORY(2A)(INFECTION# OR SYNDROME#)/TI,AB

L42 794023 S (PNEUMONIA# OR PNEUMONITIS OR PNEUMONITIDES)/TI,AB

L43 62529 S (PULMONARY OR RESPIRATORY OR LUNG)(1W)INFLAMMATION#/TI,AB

L44 188846 S RESPIRATORY INSUFFICIENCY+NT/CT OR RESPIRATORY FAILURE+NT/CT

L45 197765 S RESPIRAT?(W)(INSUFFICIENCY OR FAILURE# OR DEPRESSION OR DISTURBANCE# OR DEFICIENCY)/TI,AB

L46 131604 S RESPIRATORY DISTRESS SYNDROME+NT/CT

L47 130394 S (RESPIRAT? DISTRESS SYNDROME# OR ARDS)/TI,AB

L48 1334100 S MORBIDITY+NT/CT OR COMORBIDITY+NT/CT

L49 2144452 S (MORBIDIT? OR COMORBIDIT? OR MULTIMORBIDIT?)/TI,AB

L50 272335 S RESPIRATION, ARTIFICIAL+NT/CT OR ARTIFICIAL VENTILATION+NT/CT

L51 29354 S (ARTIFICIAL OR ASSISTED)(W)(RESPIRAT? OR VENTILATION# OR BREATHING)/TI,AB

L52 187648 S (MECHANICAL OR CONTROLLED)(W)(VENTILATION# OR RESPIRATION#)/TI,AB

L53 44187 S VENTILATORS, MECHANICAL+NT/CT OR VENTILATOR+NT/CT

L54 2111285 S (VENTILATOR# OR RESPIRATOR# OR RESPIRATORY SUPPORT# OR VENTILAT? SUPPORT#)/TI,AB

L55 94104 S OXYGEN INHALATION THERAPY+NT/CT OR OXYGEN THERAPY+NT/CT

L56 57650 S OXYGEN(W)(INHALATION# OR THERAP? OR TREATMENT# OR INSUFFLAT? OR ADMINISTRAT?)/TI,AB

L57 247360 S COST OF ILLNESS/CT OR DISEASE BURDEN+ALL/CT

L58 177676 S (BURDEN# OR COST#)(2A)(DISEASE# OR ILLNESS? OR COVID 19)/TI,AB

L59 786306 S CRITICAL CARE+NT/CT OR INTENSIVE CARE+NT/CT

L60 303974 S INTENSIVE CARE UNITS+NT/CT OR INTENSIVE CARE UNIT+NT/CT

L61 703707 S (CRITICAL OR INTENSIVE)(W)(CARE# OR THERAP? OR TREATMENT# OR UNIT# OR DEPARTMENT#)/TI,AB

L62 165 S (SUBINTENSIVE OR SUB INTENSIVE)(W)(CARE# OR THERAP? OR TREATMENT# OR UNIT# OR DEPARTMENT#)/TI,AB

L63 101377 S EMERGENCIES/CT OR EMERGENCY/CT

L64 494156 S EMERGENCY TREATMENT+NT/CT OR EMERGENCY MEDICINE+NT/CT

L65 257080 S EMERGENCY MEDICAL SERVICES+NT/CT OR EMERGENCY HEALTH SERVICE+

L66 50308 S EMERGENCY(W)(TREATMENT# OR CARE# OR THERAP# OR AID# OR HEALTH OR HEALTHCARE#)/TI,AB

L67 503545 S EMERGENCY(W)(MEDICINE OR SERVICE# OR DEPARTMENT# OR UNIT# OR ROOM#)/TI,AB

L68 158267 S (ACUTE(2W)CARE# OR PRE HOSPITAL OR PREHOSPITAL)/TI,AB

L69 19944 S (FIRST AID# OR MEDICAL URGENC? OR URGENT MEDICAL AID#)/TI,AB

L70 665858 S HOSPITALIZATION+NT/CT

L71 1370520 S (HOSPITALIZ? OR HOSPITALIS? OR HOSPITAL STAY?)/TI,AB

L72 212893 S HOSPITAL ADMISSION/CT

L73 334880 S (HOSPITAL ADMISSION# OR HOSPITAL(2A)ADMITT?)/TI,AB

L74 41899132 S L17-L73

L75 173420 S COVID-19/CT OR CORONAVIRUS DISEASE 2019/CT

L76 289288 S (COVID 19 OR COVID19 OR COVID 2019 OR COVID2019)/TI,AB

L77 62409 S (CORONAVIRUS DISEASE 2019 OR CORONA VIRUS DISEASE 2019)/TI,AB

L78 3476 S (CORONAVIRUS DISEASE 19 OR CORONA VIRUS DISEASE 19)/TI,AB

L79 75938 S SARS-COV-2/CT OR SEVERE ACUTE RESPIRATORY SYNDROME CORONAVIRUS 2+NT/CT

L80 3719 S (2019 NCOV OR 2019 N COV OR NCOV 2019 OR N COV 2019)/TI,AB

L81 4904 S (2019 NOVEL CORONAVIRUS OR 2019 NOVEL CORONA VIRUS OR NOVEL CORONAVIRUS 2019 OR NOVEL CORONA VIRUS 2019)/TI,AB

L82 55 S (2019 NEW CORONAVIRUS OR 2019 NEW CORONA VIRUS)/TI,AB

L83 628 S (CORONAVIRUS 19 OR CORONA VIRUS 19)/TI,AB

L84 3931 S (CORONAVIRUS 2019 OR CORONA VIRUS 2019)/TI,AB

L85 98306 S (SARS COV 2 OR SARS COV2)/TI,AB

L86 33965 S (SEVERE ACUTE RESPIRATORY SYNDROME CORONAVIRUS 2 OR SEVERE ACUTE RESPIRATORY SYNDROME CORONA VIRUS 2)/TI,AB

L87 40 S (SEVERE ACUTE RESPIRATORY SYNDROME CORONAVIRUS2 OR SEVERE ACUTE RESPIRATORY SYNDROME CORONA VIRUS2)/TI,AB

L88 718 S (SARS CORONAVIRUS 2 OR SARS CORONA VIRUS 2)/TI,AB

L89 81 S (WUHAN CORONAVIRUS OR WUHAN CORONA VIRUS OR WUHAN SEAFOOD MARKET PNEUMONIA VIRUS)/TI,AB

L90 339184 S L75-L89

L91 5345 S L16 AND L74 AND L90

L92 5327 S L91 AND (ENGLISH OR FRENCH OR SPANISH OR ITALIAN)/LA

L93 5321 S L92 AND 2020-2021/PY

L94 3276 DUP REM L93 (2045 DUPLICATES REMOVED)

**sAppendix 3: Search strategy**

**Definition of “Migrant”’**:

We referred to the definition described in the IOM glossary: “An umbrella term, not defined under international law, reflecting the common lay understanding of a person who moves away from his or her place of usual residence, whether within a country or across an international border, temporarily or permanently, and for a variety of reasons. The term includes a number of well-defined legal categories of people, such as migrant workers; persons whose particular types of movements are legally defined, such as smuggled migrants; as well as those whose status or means of movement are not specifically defined under international law, such as international students”.

**Definition of “refugee”:**

We referred to the definition from the convention and protocol relating to the status of refugees of the United Nations High Commissioner for Refugees: (Any person who) “As a result of events occurring before 1 January 1951 and owing to well-founded fear of being persecuted for reasons of race, religion, nationality, membership of a particular social group or political opinion, is outside the country of his nationality and is unable or, owing to such fear, is unwilling to avail himself of the protection of that country; or who, not having a nationality and being outside the country of his former habitual residence as a result of such events, is unable or, owing to such fear, is unwilling to return to it”.

**Definition of “ethnic minority”:**

We referred to the definition of the European Centre for Disease Prevention and Control: “Groups of people sharing a different ‘sense of identity and common characteristics such as language, religion, tribe, nationality, race or a combination thereof’ from the majority population in the place where they live”.
